# P53 Trends in antimicrobial resistance and antibiotic utilization in England: a 5 year analysis of ESPAUR surveillance data (2019–24)

**DOI:** 10.1093/jacamr/dlag102.059

**Published:** 2026-06-26

**Authors:** Ada Cernaliu, Rasha Abdelsalam Elshenawy

**Affiliations:** School of Health, Medicine and Life Sciences, University of Hertfordshire, Hatfield, UK; School of Health, Medicine and Life Sciences, University of Hertfordshire, Hatfield, UK

## Abstract

**Background:**

Antimicrobial resistance (AMR) poses a growing public health threat in England, contributing to significant morbidity, mortality and healthcare system pressure. The UK Health Security Agency (UKHSA) English Surveillance Programme for Antimicrobial Utilisation and Resistance (ESPAUR) monitors antibiotic use and resistance across care settings.^1^ The COVID-19 pandemic disrupted prescribing patterns, reducing primary care use while increasing hospital prescribing due to clinical uncertainty.^2^ This study analyses AMR and prescribing trends from 2019 to 2024, assessing WHO AWaRe alignment and National Action Plan targets.

**Objectives:**

This study aimed to track changes in resistant bloodstream infection rates for *Escherichia coli*, *Klebsiella pneumoniae* and *Staphylococcus aureus*, compare prescribing patterns across primary and secondary care, and assess alignment with WHO AWaRe guidelines.

**Methods:**

A secondary data analysis was carried out using publicly available data from the UKHSA ESPAUR Report 2019–2024. Resistant BSI rates per 100 000 population, antibiotic consumption (defined daily doses per 1000 inhabitants per day; DID) and AWaRe prescribing proportions were extracted for 2019–24. Descriptive statistics and percentage change calculations were used to identify trends. Ethical approval was not needed, as all data were publicly available and anonymized.

**Results:**

Resistant bloodstream infection rates showed a temporary decline during the COVID-19 pandemic before exceeding 2019 baseline levels by 2024. *E. coli* rates increased from 23.5 to 25.0 per 100 000 population (+6.4%), *K. pneumoniae* from 3.6 to 4.9 per 100 000 (+36.1%), and *S. aureus* from 1.4 to 1.8 per 100 000 (+28.6%). With respect to antibiotic prescribing volumes, primary care GP prescribing showed an overall reduction from 12.862 to 12.339 DID, while hospital inpatient prescribing rose modestly above its 2019 baseline from 2.344 to 2.409 DID; total antibiotic use in 2024 was 17.53 DID, marginally exceeding the NAP target of less than or equal to 16.92 DID. Regarding AWaRe alignment, Access antibiotic prescribing improved in primary care, increasing from 63.2% to 65.6% over the study period. However, hospital inpatient Access proportions remained consistently lower, rising only from 53.3% to 54.0%, with Watch antibiotic use in hospital settings (42.7% in 2024) exceeding that of primary care by 13 percentage points. England achieved two of four NAP 2019–24 targets: the greater than or equal to 60% Access prescribing threshold and an estimated greater than or equal to 10% reduction in Watch and Reserve antibiotic use. Hospital settings were identified as the priority area for further targeted antimicrobial stewardship interventions.

**Conclusions:**

England has demonstrated measurable progress in antimicrobial stewardship, particularly through improved Access antibiotic prescribing in primary care. The ESPAUR surveillance framework, led by UKHSA, provides a robust and actionable evidence base to build upon. To sustain and accelerate this momentum, it is recommended that stewardship programmes are prioritized in secondary care, Access antibiotic prescribing in hospital settings is increased, and UKHSA-led national surveillance capacity is further strengthened. Continued investment in ESPAUR-informed stewardship initiatives presents a clear and evidence-based opportunity to fully achieve NAP 2024–29 targets and substantially reduce the AMR burden across England.

## References

[1] UKHSA . English Surveillance Programme for Antimicrobial Utilisation and Resistance (ESPAUR) Report. GOV.UK. 2014. https://www.gov.uk/government/publications/english-surveillance-programme-antimicrobial-utilisation-and-resistance-espaur-report

[2] Abdelsalam Elshenawy R, Umaru N, Aslanpour Z. WHO AWaRe classification for antibiotic stewardship: tackling antimicrobial resistance – a descriptive study from an English NHS Foundation Trust prior to and during the COVID-19 pandemic. Front Microbiol 2023; 14: 1298858. 38146447 10.3389/fmicb.2023.1298858 PMC10749484

